# Risk of sexually transmitted infections following depressive disorder: A nationwide population-based cohort study

**DOI:** 10.1097/MD.0000000000012539

**Published:** 2018-10-26

**Authors:** Sheng-Yun Huang, Jeng-Hsiu Hung, Li-Yu Hu, Min-Wei Huang, Shyh-Chyang Lee, Cheng-Che Shen

**Affiliations:** a Department of Psychiatry, Taichung Veterans General Hospital, Taichung; b Department of Psychiatry, Chiayi Branch, Taichung Veterans General Hospital, Chiayi; c Department of Obstetrics and Gynecology, Taipei Tzu Chi Hospital, Buddhist Tzu Chi Medical Foundation, Taipei; d School of Medicine, Tzu Chi University, Hualien; e Department of Psychiatry, Taipei Veterans General Hospital; f School of Medicine, National Yang-Ming University, Taipei; g School of Medicine, China Medical University, Taichung; h Department of Information Management, Chia Nan University of Pharmacy & Science, Tainan; i Department of Orthopedics, Chiayi Branch, Taichung Veterans General Hospital; j Center for Innovative Research on Aging Society, National Chung Cheng University, Chiayi, Taiwan.

**Keywords:** chlamydia, depression, gonorrhoeae, human immunodeficiency virus, sexually transmitted infections, syphilis, warts

## Abstract

Depressive disorder is a severe mental disorder associated with functional and cognitive impairment. Numerous papers in the literature investigated associations between sexually transmitted infections (STIs) and psychiatric illnesses. However, the results of these studies are controversial.

We explored the relationship between depressive disorder and the subsequent development of STIs including human immunodeficiency virus (HIV) infection, primary, secondary, and latent syphilis, genital warts, gonorrhea, chlamydial infection, and trichomoniasis.

We identified patients who were diagnosed with the depressive disorder in the Taiwan National Health Insurance Research Database. A comparison cohort was constructed of patients without the depressive disorder who were matched according to age and sex. The occurrence of subsequent new-onset STIs was evaluated in both cohorts.

The depression cohort consisted of 5959 patients, and the comparison cohort consisted of 23,836 matched control patients without depressive disorder. The incidence of subsequent STIs (hazard ratio [HR] 1.54, 95% confidence interval [CI] 1.34–1.76) was higher among the depressed patients than among the patients in the comparison cohort. Furthermore, female gender compared to male (HR 1.58, 95% CI 1.24–2.01) and young age <40-year-old (HR 1.79, 95% CI 1.38–2.32) are both risk factors for acquisition of STIs in depression patient. For individual STI, the results indicated that the patients with depressive disorder exhibited a markedly higher risk for subsequent STIs including HIV infection, syphilis, genital warts, gonorrhea, chlamydial infection, and trichomoniasis.

Depressive disorder might increase the risk of subsequent newly diagnosed STIs including HIV infection, syphilis, genital warts, gonorrhea, chlamydial infection, and trichomoniasis in Taiwan population. Clinicians should pay particular attention to STIs in depression patients. Depression patients, especially those with the history of high-risk sexual behaviors, should be routinely screened for STIs.

## 1 Introduction

The depressive disorder is a commonly occurring, severe and recurrent disorder linked to diminished role functioning and quality of life, medical morbidity, and mortality.^[1]^ Convergent evidence indicates that depressive disorder is one of the leading cause of disability among patients in both developed and emerging economies.^[2]^ The principal source of cost and illness-associated morbidity is due to a significant decrease in role-function among affected individuals.^[3,4]^ As for the relationships between depression and sexually transmitted infections (STIs), depression may impair cognitive function and memory,^[5]^ decrease impulse control,^[6]^ which contribute to risky sexual practices. These depression-related effects may inhibit the clear perception of STIs risk and the ability to prevent risk behavior.^[7,8]^

The STIs are infections that are commonly spread by sexual behaviors. In 2015, about 1.1 billion people had STIs other than human immunodeficiency virus (HIV) infection.^[9]^ STIs other than HIV infection resulted in 108,000 deaths in 2015.^[10]^ In the United States, millions of cases of STIs occur each year, resulting in substantial medical costs to the nation. A study indicated that the total lifetime direct medical cost for STIs in 2008 in the United States was $15.6 billion and the burden of STIs would be even greater in the absence of STIs prevention and control efforts.^[11]^ Therefore, it is essential to identify risk factors of STIs and arrange appropriate screening protocols for people with risk factors.

Numerous papers in the literature investigated associations between STIs and depressive disorders.^[7,8,12–20]^ While STIs is risk factors for depression, depression also may increase susceptibility to risk behaviors and infection.^[7,14,18–20]^ However, most of these studies focused on HIV infection only^[13,15,18,20]^ and few studies were designed to investigate the association between depressive disorder and other STIs, such as syphilis, genital warts, gonorrhea,^[5,17]^ chlamydial infection,^[5,8]^ and trichomoniasis.^[5,17]^ In addition, the results of these studies are controversial. For example, a study investigated of 10,783 young adults in the United States revealed that depression was associated with increased risk of STIs only among black men and there is no significantly higher risk among women.^[17]^ However, another study invested similar group with 18,142 cases in the United States found that a major depressive episode was associated with increased risk of STIs only in females.^[16]^ Furthermore, most of these study results are based on cross-sectional study design and lack a longitudinal perspective.^[8,9,13,16,18,20,21]^

In response to the few longitudinal studies concerning the association between depressive disorder and the subsequent risk of STIs, and based on the hypothesis that depressive disorder, a psychiatric illness related to impaired cognitive function and poor impulse control, might have a higher risk for developing subsequent STIs, we designed a nationwide population-based retrospective cohort study to investigate the possible link between these 2 illnesses.

## 2 Patients and methods

### 2.1 Data sources

Instituted in 1995, the National Health Insurance (NHI) program is a mandatory health insurance program that offers comprehensive medical care coverage, including outpatient, inpatient, emergency, and traditional Chinese medicine, to all residents of Taiwan, with a coverage rate of up to 98%. The NHI Research Database (NHIRD) contains comprehensive information regarding clinical visits, including prescription details and diagnostic codes based on the A code and International Classification of Diseases, Ninth Revision, Clinical Modification (ICD-9-CM). The NHIRD is managed and publicly released by the National Health Research Institute (NHRI) for research purposes, and confidentiality is maintained according to the directives of the Bureau of NHI. The data source for our study was the Longitudinal Health Insurance Database 2000 (LHID 2000), which is a data set of NHIRD. Data for the LHID were collected systematically and randomly sampling from the NHIRD; the database included the data of 1 million individuals. The NHRI of Taiwan reports that there were no significant differences in gender distribution, age distribution, or average insured payroll-related amount between the patients in the LHID and those in the original NHIRD.

### 2.2 Ethics statement

The Institutional Review Board of Taichung Veterans General Hospital approved this study. Written consent from the study patients was not obtained, because the NHI data set consists of de-identified secondary data for research purposes and the Institutional Review Board of Taipei Veterans General Hospital issued a formal written waiver for the need for consent.

### 2.3 Study population

Using data extracted from the LHID 2000, we conducted a retrospective cohort study of patients who were newly diagnosed with depressive disorder between January 1, 2000 and December 31, 2004. The patients with the depressive disorder were defined as ICD-9-CM code: 296.2, 296.3, 300.1, and 311. We excluded patients who were diagnosed with depressive disorder between January 1, 1996 and December 31, 1999. We also excluded patients who were diagnosed with STIs (HIV infection, primary, secondary, and latent syphilis, genital warts, gonorrhea, chlamydial infection, and trichomoniasis) before they were diagnosed with depressive disorder. For each depressive patient included in the final cohort, 4 age- and sex-matched control patients without STIs were randomly selected from the LHID 2000. The random assignment procedures were performed by SAS statistical software and were based on the random numbers which were generated from the uniform distribution. All depression and control patients were observed until diagnosed with HIV infection (ICD-9-CM codes: 042 or V08, A049), primary, secondary, and latent syphilis (ICD-9-CM codes: 091-097, A060), genital warts (ICD-9-CM codes: 078.11), gonorrhea (ICD-9-CM codes: 098, A061), chlamydial infection (ICD-9-CM codes: 078.8, 078.88), or trichomoniasis (ICD-9-CM codes: 131, A079), or until death, withdrawal from the NHI system, or December 31, 2013. The primary clinical outcomes assessed were newly diagnosed HIV infection, primary, secondary, and latent syphilis, genital warts, gonorrhea, chlamydial infection, and trichomoniasis. Common comorbidities including hypertension, diabetes mellitus, dyslipidemia, coronary artery diseases, congestive heart failure, chronic pulmonary diseases, and cerebrovascular diseases were also compared between depression and control patients.

### 2.4 Statistical analysis

The occurrence of newly diagnosed HIV infection, primary, secondary, and latent syphilis, genital warts, gonorrhea, chlamydial infection, or trichomoniasis in the depression and control patients were considered as the primary outcome in this study. We first compared the distribution of demographic characteristics between the depression and control patients by using the independent *t* tests and Chi-squared test. To investigate potential surveillance bias, subgroups were stratified according to the duration since enrollment. In addition, a Cox proportional-hazards regression model was performed to identify the risk factors associated with STIs in the whole sample and patients with depressive disorder. Furthermore, the Cox proportional-hazards regression model was also constructed to calculate the hazard ratio (HR) of 6 different kinds of STIs, including HIV infection, primary, secondary, and latent syphilis, genital warts, gonorrhea, chlamydial infection, and trichomoniasis of the depression cohort and control cohort.

The SAS statistical software for Windows, Version 9.3 (SAS Institute, Cary, NC), was used for data extraction, computation, linkage, processing, and sampling. All other statistical analyses were performed using the SPSS statistical software for Windows, Version 20 (IBM, Armonk, NY). The results of comparisons with a *P*-value <.05 were considered to indicate a statistically significant relationship.

## 3 Results

Our study sample comprised 5959 depression patients and 23,836 control patients without depressive disorder. The comparisons of the demographic and clinical variables between the depression and control patients are presented in Table 1. The median age of the patients was 42.6 years (interquartile range, 29.5–52.4 years) and the median follow-up duration of depression and control cohorts was 11.08 and 11.36 years, respectively. A higher percentage of patients with the depressive disorder were observed in the group of people aged 20 to 39 years. Baseline difference in comorbidities demonstrated the higher prevalence of hypertension, diabetes mellitus, dyslipidemia, coronary artery diseases, congestive heart failure, chronic pulmonary diseases, and cerebrovascular diseases among the depressed patients. During the follow-up period, 298 (5.0%) depression patients and 774 (3.2%) control patients were diagnosed with STIs (*P* < .001). The most common subsequent STIs in the patients with the depressive disorder were trichomoniasis in 121 patients (2.0%) and chlamydial infection in 68 patients (1.1%). Overall, significantly higher incidences of all STIs included HIV infection (*P* = .001), syphilis (*P* < .001), genital warts (*P* = .001), gonorrhea (*P* < .001), chlamydial infection (*P* = .006), and trichomoniasis (*P* = .004) were observed in the depression patients than in the control patients.

**Table 1:**
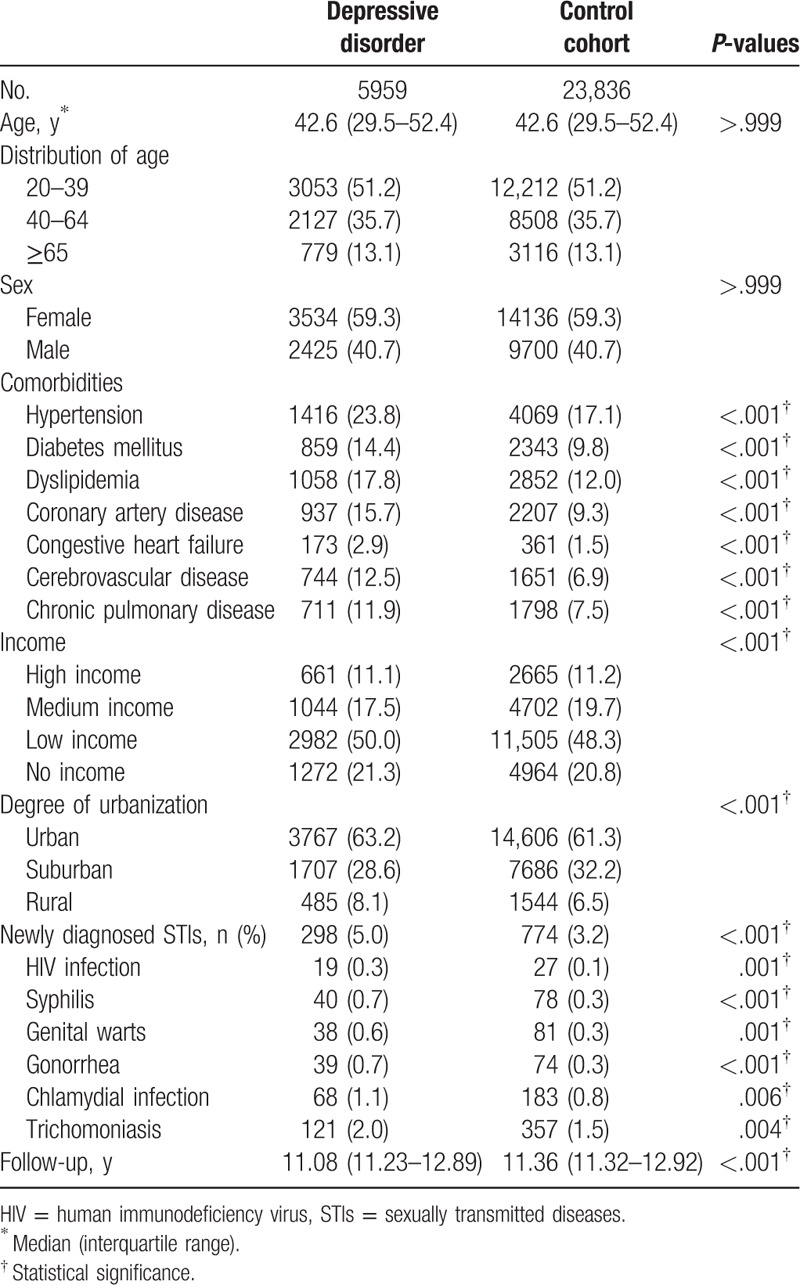
Characteristics of depressive disorder and sexually transmitted diseases (STIs) and control subjects.

A subanalysis based on the duration of follow-up revealed that most of the STIs developed beyond the first year following a depression diagnosis and that the risk of all STIs included HIV infection, syphilis, genital warts, gonorrhea, chlamydial infection, and trichomoniasis remained significantly elevated when the patients diagnosed with STIs within 1 year of depression diagnosis were excluded. The results of the subanalysis are summarized in Table 2.

**Table 2:**
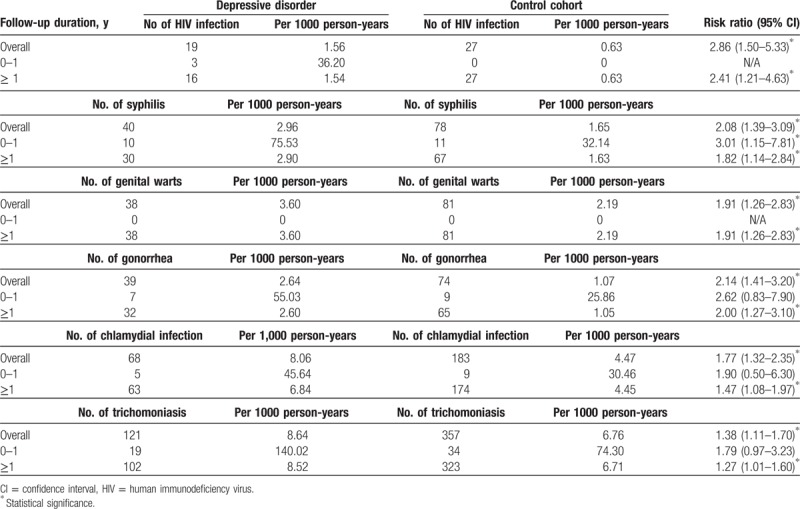
Number of newly diagnosed sexually transmitted diseases between depressive disorder and control subjects which were stratified by follow-up duration.

Besides, a Cox proportional-hazards regression analysis was conducted to calculate the HR of the newly diagnosed STIs for the patients with the depressive disorder compared with the matched controls and revealed patients with depressive disorder exhibited a markedly higher risk for subsequent STIs (HR 1.54, 95% confidence interval [CI] 1.34–1.76). In addition, female gender compared to male (HR 2.20, 95% CI 1.91–2.54), young age <40 year old (HR 1.69, 95% CI 1.48–1.92), cerebrovascular disease (HR 1.42, 95% CI 1.14–1.78), and low income (HR 1.22, 95% CI 1.05–1.43) are risk factors for acquisition of STIs. Urbanization and other comorbidities including hypertension, diabetes mellitus, dyslipidemia, coronary artery diseases, congestive heart failure, and chronic pulmonary diseases are not risk factors for the subsequent STIs (Table 3).

**Table 3:**
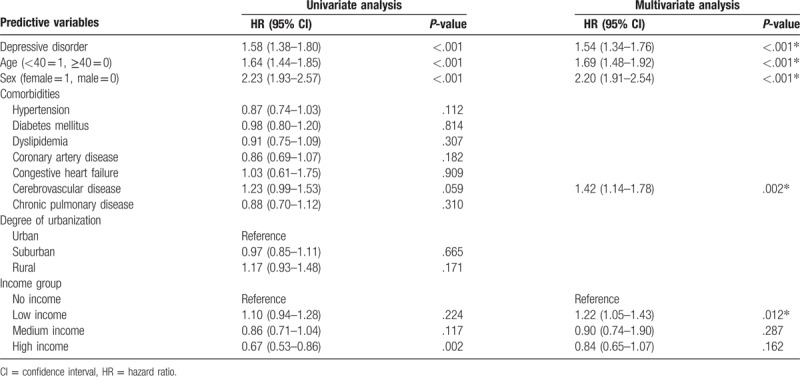
Analyses of risk factors for sexually transmitted diseases in patients with and without depressive disorder.

In depression patients, female gender compared to male (HR 1.58, 95% CI 1.24–2.01) and young age less than the age of 40 (HR 1.79, 95% CI 1.38–2.32) are both risk factors for acquisition of STIs (Table 4). For individual STI, the results indicated that the patients with depressive disorder exhibited a markedly higher risk for subsequent STIs included HIV infection (HR 2.93, 95% CI 1.62–5.30), syphilis (HR 1.95, 95% CI 1.32–2.88), genital warts (HR 1.88, 95% CI 1.27–2.77), gonorrhea (HR 1.97, 95% CI 1.33–2.92), chlamydial infection (HR 1.44, 95% CI 1.08–1.90), and trichomoniasis (HR 1.33, 95% CI 1.08–1.64) (Table 5).

**Table 4:**
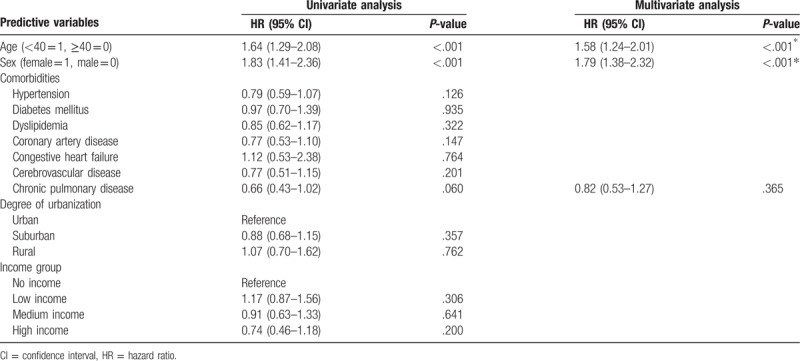
Analyses of risk factors for sexually transmitted diseases in patients with depressive disorder.

**Table 5:**
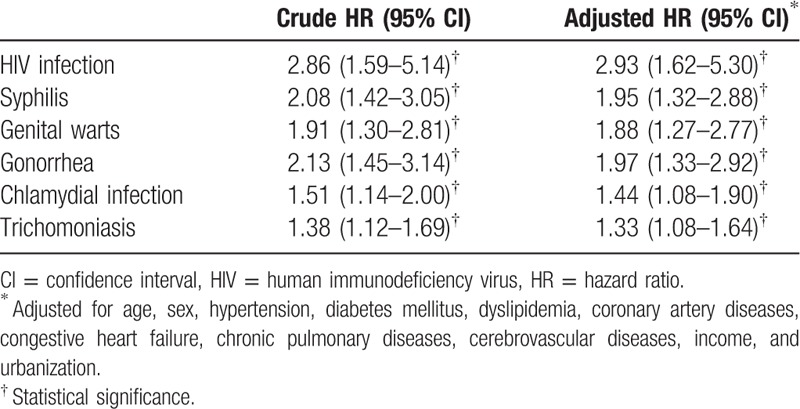
Hazard ratios of time until sexually transmitted diseases between depressive disorder and control subjects during a 10-year follow-up period.

## 4 Discussion

The key findings of our study are listed as below: the prevalence of STIs, including HIV infection, primary, secondary, and latent syphilis, genital warts, gonorrhea, chlamydial infection, and trichomoniasis, in patients with depressive disorder were demonstrated in our work; depressive disorder was significantly associated with an increased sequential risk of all STIs included HIV infection, syphilis, genital warts, gonorrhea, chlamydial infection, and trichomoniasis in Taiwan population; female gender compared to male (HR 1.58) and young age <40 year old (HR 1.79) are both risk factor for acquisition of STIs in depression patients.

In our study, it revealed that depressive disorder was significantly associated with an increased sequential risk of STIs in Taiwan population. There are several possible explanations for the increased risk of STIs in patients with depressive disorder. First, depression may influence their ability to negotiate and engage in safer sexual behavior.^[7]^ Depression patients also lack adequate self-esteem and the assertiveness skills needed to implement safer sex practices.^[8]^ Second, the impaired cognitive function was noted in depression, and it may result in neglect of risky sexual behavior. Depressive symptoms may also be linked to poor sexual decision-making and make them engage in risky sexual practices.^[5]^ Third, depressed patients were more likely to have sex for money or drugs, to have had sex with an intravenous drug user, to have early sex age, and to have a greater number of lifetime sex partners.^[15,21]^ Also, the population of depression patient is in a more complicated environment of sexual initiation, continued sexual activity, choice and number of partners, and contextual prevalence of STD.^[16]^ Finally, substance use disorders are common comorbidities in patients with depressive disorder^[22]^ and previous works have demonstrated that substance abuse and dependence increase the risk of STIs.^[23–25]^

Reviewing the previous study, most of the research focuses on the association between depression and risky sexual behavior^[15,21]^ which lack evidence about the relationships of biologically verified STIs. As for our study, we demonstrated the risk of biologically diagnosed STIs following depressive disorder. Besides, the results of past research are controversial, some revealed increased risk of STIs only among depression male,^[17]^ some revealed the increased risk of STIs only among depression female^[7,16]^ and even some showed no growing risk among depression patient.^[13]^ Reviewing their research method, most of the study focus on the specific groups such as STIs clinical patient,^[15]^ military,^[13]^ black race,^[7,26]^ or Homeless adolescent.^[8]^ The controversial result may be due to the selection of different groups and our study survey the general population of Taiwan which is more extensive than those studies.

In the present study, we conducted a subgroup analysis stratified according to the duration between the diagnosis of depressive disorder and new-onset STIs (Table 2). The results indicated that most STIs included HIV infection, genital warts, gonorrhea, chlamydial infection, and trichomoniasis were increased beyond the first year following a depression diagnosis. Patients with depressive disorder are likely to exhibit a higher frequency of outpatient and inpatient visits than the general population, leading to an earlier diagnosis of STIs which cause surveillance bias. However, in our work, the risk ratio for the newly diagnosed HIV infection, genital warts, gonorrhea, chlamydial infection, and trichomoniasis did not reach statistically significant within 1 year of depression diagnosis for the depression cohort. Moreover, when patients diagnosed with STIs within 1 year of depression diagnosis were excluded, the risk ratio for the all newly diagnosed STIs included HIV infection, syphilis, genital warts, gonorrhea, chlamydial infection, and trichomoniasis remained high for the depression cohort, and the ratios were all statistically significant. Thus, this result suggests that the increased risks of HIV infection, genital warts, gonorrhea, chlamydial infection, and trichomoniasis in depression patients were not caused by surveillance bias.

Our study revealed that female gender compared to male (HR 1.58) and young age <40 years old (HR 1.79) are both risk factors for depression patients to acquire STIs. There are some possible reasons that female gender is a risk factor for STIs in depression patient. According to the previous study, higher rates of sexual risk behaviors in female depression patients than male depression patients was noted.^[16,21]^ Furthermore, a lack of female-controlled protective devices^[27]^ and the initial phase of STIs is often asymptomatic in women^[28]^ also add to the vulnerability of these patients. As for young age, there are several studies discussed risk factor of STIs in general population and age is one of the significant risk factors for STIs.^[29,30]^ Young people are at high risk of infection because they may be more vulnerable biologically, lack the social skills to negotiate safe sex, and they lack appropriate health care delivery.^[28]^

Consistent with previous studies,^[31,32]^ in our work, it revealed that low income (HR 1.22) was significantly associated with an increased sequential risk of STIs. However, we also found that cerebrovascular disease (HR 1.42) was a risk factor of STIs. No epidemiologic studies have investigated the association between cerebrovascular disease and STIs. Further prospective clinical studies on the relationship between STIs and cerebrovascular disease are warranted.

There are several strengths of our study. First, our study design included an unbiased patient selection process. Because participation in the NHI is mandatory and all residents of Taiwan can access healthcare with low copayments, referral bias is low, and follow-up compliance is high. Second, our study is a population-based study including large sample sizes from all hospitals in the country. With small samples or single hospital case studies popular in existing literature, it is difficult to develop such an index with a greater acceptance across the healthcare industry. Third, the data used in the study were derived from the National Health Insurance system in Taiwan. As an observational database, these data reflect current real-world diagnostic patterns.

However, it has several limitations inherent to the use of claims databases that should be considered. First, the NHIRD does not provide detailed information on patients such as numbers of sex partners, sex with promiscuous partners, and condom use. They are all major risk factors for STIs. Thus, we were unable to control for these potentially confounding factors. Second, compared with previous studies regarding the prevalence of STIs in patients with mental disorders, the prevalence of STIs in our work is relatively lower. For examples, in a study surveying the prevalence of STIs in psychiatric outpatients, the results showed the prevalence of gonorrhea was 1%, chlamydial infection 3.3%, and T trichomoniasis 15.7%.^[33]^ Underestimate of the prevalence of STIs in our work should be taken into consideration. Because STIs may be asymptomatic, some patients with STIs may not search for medical treatment and were not diagnosed. Moreover, STI patients may felt stigmatized because of their STIs and did not want to search for medical help. Third, the diagnostic accuracy and severity of the depressive disorder in our work could not be obtained. Whether the severity of the depressive disorder is related to STIs development should be further evaluated.

In conclusion, this nationwide cohort study indicated an increased risk of HIV infection, syphilis, genital warts, gonorrhea, chlamydial infection, and trichomoniasis among depression patients in Taiwan. Female gender and young age are both risk factor for acquisition of STIs in depression patients. Taking a sexual history in persons with the depressive disorder is, and those engaging in high-risk behavior should be routinely screened for STIs allowing for detection, treatment, and preventive education. Furthermore, prospective studies, particularly those with additional patient-level data, are warranted to confirm our findings.

## Acknowledgment

The authors thank the Research Center of Medical informatics at Kaohsiung Veterans General Hospital for statistical assistance. The study is based on data from the National Health Insurance Research Database provided by the Bureau of National Health Insurance (BNHI) in Taiwan and managed by National Health Research Institutes (NHRI). The authors express our particular gratitude to the government organization BNHI and the nonprofit foundation NHRI.

## Author contributions

**Conceptualization:** Sheng-Yun Huang, Shyh-Chyang Lee, Cheng-Che Shen.

**Data curation:** Jeng-Hsiu Hung.

**Formal analysis:** Jeng-Hsiu Hung, Li-Yu Hu.

**Investigation:** Li-Yu Hu, Min-Wei Huang.

**Methodology:** Jeng-Hsiu Hung.

**Resources:** Min-Wei Huang.

**Supervision:** Min-Wei Huang, Shyh-Chyang Lee.

**Writing – original draft:** Sheng-Yun Huang.

**Writing – review & editing:** Min-Wei Huang, Cheng-Che Shen.

Cheng-Che Shen orcid: 0000-0002-8080-2841.
